# Influence of bilateral nasal packing on sleep oxygen saturation after general anesthesia: A prospective cohort study

**DOI:** 10.3389/fsurg.2023.1083961

**Published:** 2023-01-30

**Authors:** Shuwen Guan, Tingting Zhao, Jingying Ye, Junbo Zhang

**Affiliations:** ^1^ Department of Otolaryngology, Head and Neck Surgery, Peking University First Hospital, Beijing, China; ^2^ Department of Otorhinolaryngology, Head and Neck Surgery, Shenzhen University General Hospital, Shenzhen, China; ^3^ Department of Head and Neck Surgery, National Cancer Center/National Clinical Research Center for Cancer/Cancer Hospital & Shenzhen Hospital, Chinese Academy of Medical Sciences and Peking Union Medical College, Shenzhen, China; ^4^ Department of Otolaryngology, Head and Neck Surgery, Beijing Tsinghua Changgung Hospital, School of Clinical Medicine, Tsinghua University, Beijing, China

**Keywords:** bilateral nasal packing, sleep oxygen saturation, first night after general anesthesia, operation on nasal cavity, a prospective cohort study

## Abstract

**Objective:**

This study aims to evaluate the effect of bilateral nasal packing on sleep oxygen saturation and its influencing factors on the first night after general anesthesia.

**Method:**

A total of 36 adult patients who underwent bilateral nasal packing with a nonabsorbable expanding sponge after general anesthesia surgery were prospectively studied. All these patients underwent overnight oximetry tests before and the first night after surgery. The following oximetry variables were collected for analysis: the lowest oxygen saturation (LSAT), the average oxygen saturation (ASAT), the oxygen desaturation index of ≥4% (ODI4), and the percentage of time with oxygen saturation below 90% (CT90).

**Results:**

Among the 36 patients, the incidences of both sleep hypoxemia and moderate-to-severe sleep hypoxemia increased with bilateral nasal packing after general anesthesia surgery. All the pulse oximetry variables we studied deteriorated significantly after surgery: both LSAT and ASAT decreased significantly (*P* < 0.05), while both ODI4 and CT90 increased significantly (*P* < 0.05). In a multiple logistic regression analysis, body mass index (BMI), LSAT, and modified Mallampati grade were found to be independently predictive for a larger decrease in LSAT (≥5%) after surgery (all *P*’s < 0.05).

**Conclusion:**

Bilateral nasal packing after general anesthesia could induce or aggravate sleep hypoxemia, especially in patients with obesity, relatively normal sleep oxygen saturation, and high modified Mallampati grades.

## Introduction

Bilateral nasal packing is common management after nasal septum surgery, which aims to reduce the possibility of complications such as bleeding, nasal adhesion, and nasal septum hematoma. Furthermore, it is believed to stabilize the septum to reduce the risk of postoperative deviation during the recovery period (1). However, its use has been controversial for the past decades. Compared with alternative methods, especially trans-septal sutures, bilateral nasal packing has a tendency to cause postoperative pain, infection, crusting, epiphora, and dysphagia (2–4). On the other hand, such patients are more likely to develop or worsen sleep-disordered breathing during the early postoperative nights due to unstable oral breathing and the accumulation of anesthetic drugs, especially the first postoperative night, which could increase the risks of respiratory and cardiovascular complications.

In the current study, the changes in sleep oxygen saturation and its influencing factors before and the first night after septoplasty under general anesthesia were prospectively studied in a group of patients with postoperative bilateral nasal packing. The results may be a valuable reference for identifying patients at high risk of complications related to severe deterioration of sleep hypoxemia.

## Materials and methods

### Study population

Between January 2022 and June 2022, a total of 36 consecutive patients who successfully underwent septoplasty alone or septoplasty combined with functional endoscopic sinus surgery in Peking University First Hospital were prospectively studied. The detailed inclusion criteria are as follows: (1) age ≥18 years old; (2) bilateral nasal packing after surgery with a nonabsorbable expanding sponge (Merocel; Medtronic Xomed, Jacksonville, FL, USA); (3) no simultaneous pharyngeal operation; (4) similar anesthetic drugs, including midazolam, sufentanil, and propofol for induction of anesthesia, propofol for maintenance of anesthesia, and rocuronium bromide for muscle relaxation; and (5) no severe coexisting cardiovascular, pulmonary, or neuropsychiatric disorders. All these patients underwent an overnight oximetry test before and the first night after surgery. Other postoperative managements that may affect sleep oxygen saturation were consistent in all patients, including (1) no supplemental analgesic agents after recovery from general anesthesia; (2) no oxygen inhalation during the first postoperative night; and (3) no elevation of the head of the bed. This study has been approved by the Ethics Committee of Peking University First Hospital, and all patients have provided signed informed consent.

### Overnight pulse oxygen monitoring

Before and the first night after surgery, all the overnight oximetry tests were performed using a pulse oximeter (Orange Family Technology, Tianjin, China). The oximeter was turned on before going to bed and was closed after waking up on the second morning. The primary data were analyzed by supporting software, and the results were rechecked by an experienced technician to obtain the following variables: the lowest oxygen saturation (LSAT), the average oxygen saturation (ASAT), the oxygen desaturation index of ≥4% (ODI4), and the percentage of time with oxygen saturation below 90% (CT90).

### Clinical data collection

The same physician collected all the variables for analysis after the completion of the postoperative oximetry test, including age, sex, body mass index (BMI), tonsil grade, palate position, minimal cross-sectional area of the pharynx (MCAP), anesthesia duration, and recovery time. In this study, BMI was calculated as weight (kg)/height (m)^2^, the tonsil grade was determined by the Brodsky Grading Scale (5), the palate position was determined by the modified Mallampati scale (6), the MCAP was measured after reviewing all axial CT images from the nasopharynx to the hypopharynx, and the recovery time was determined as the time duration between the end of anesthesia and the start of sleep on the day of surgery.

### Statistical analysis

In this study, standard statistical procedures were carried out using SPSS 20.0 (IBM, Armonk, NY, USA). All continuous variables were displayed as mean value ± standard deviation. The paired Student *t*-test was used to compare preoperative and postoperative mean values. The unpaired Student *t*-test was used to compare continuous variables between different groups. The Pearson chi-square test was used to compare categorical variables between different groups. Multiple logistic regression analysis was used to evaluate the significance of independent variables. The cutoff point for all statistical significance was *P* < 0.05.

## Results

**Figure 1:**
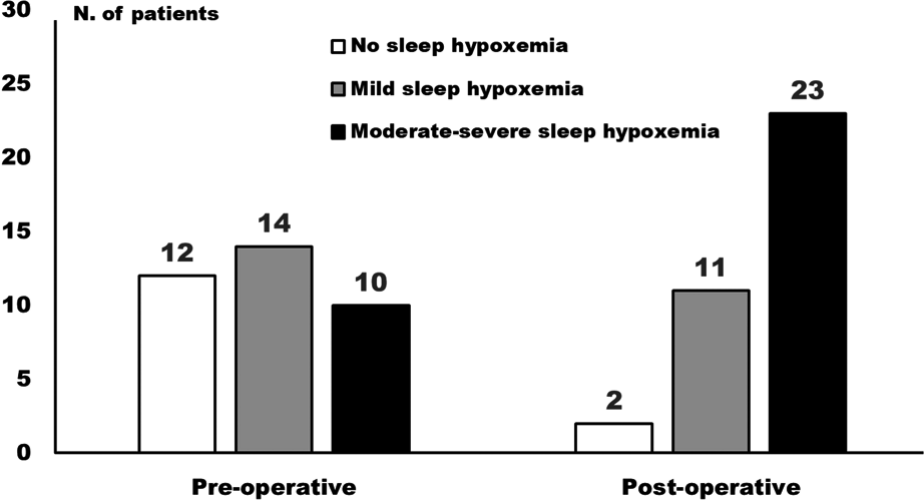
Distribution of patients with no, mild, and moderate-to-severe sleep hypoxemia before and the night after the operation.

All 36 patients had successfully completed the overnight pulse oxygen tests before and the first night after surgery. In the current study, mild sleep hypoxemia was determined as the LSAT of <90% but ≥85%, while moderate-to-severe sleep hypoxemia was determined as the LSAT of <85%. The number of patients with no, mild, and moderate-to-severe sleep hypoxemia before and the first night after surgery is shown in Figure 1, indicating obvious increases in the incidences of both sleep hypoxemia and moderate-to-severe sleep hypoxemia. In addition, as shown in Table 1, all pulse oximetry variables we collected deteriorated significantly after surgery: both LSAT and ASAT decreased significantly (*P* < 0.05), while both ODI4 and CT90 increased significantly (*P* < 0.05).

**Table 1:** Changes of pulse oximetry variables after surgery in the 36 patients with bilateral nasal packing.

	Before	After	*P*-value
LSAT (%)	85.4 ± 7.1	81.8 ± 6.4	<0.001*
ASAT (%)	94.5 ± 1.8	93.2 ± 2.2	<0.001*
ODI4 (events/h)	10.9 ± 10.8	27.1 ± 21.4	<0.001*
CT90 (%)	5.6 ± 14.0	14.1 ± 22.7	0.012*

LSAT, lowest oxygen saturation; ASAT, average oxygen saturation; ODI4, oxygen desaturation index of ≥4%; CT90, and percentage of time with oxygen saturation below 90%.

* Statistically significant.

A cutoff value of 5% for LSAT difference (ΔLSAT) calculated by preoperative LSAT minus postoperative LSAT was used to group all 36 patients. The numbers of patients with ΔLSAT ≥5% and ΔLSAT < 5% were 15 (41.7%) and 21 (58.3%), respectively. A comparison of baseline information, including preoperative pulse oximetry variables, between these two groups is presented in Table 2: the preoperative LSAT was significantly higher in patients with ΔLSAT ≥5% than in patients with ΔLSAT < 5% (*P* = 0.010), while no other variables differed significantly between these two groups (all *P*’s > 0.05).

**Table 2:** Comparison of baseline information and pulse oximetry variables of patients in different ΔLSAT groups after bilateral nasal packing.

	All patients (*n* = 36)	ΔLSAT ≥ 5% (*n* = 15)	ΔLSAT < 5% (*n* = 21)	*P*-value
Sex				0.650
Male	30	13	17	
Female	6	2	4	
Age (years)	39.2 ± 14.0	40.0 ± 13.2	38.6 ± 14.9	0.786
BMI (kg/m^2^)	25.9 ± 4.3	27.0 ± 5.0	24.8 ± 3.6	0.132
Tonsil grade				0.463
I	19	9	10	
II–III	17	6	11	
Modified Mallampati grade				0.473
I–II	24	9	15	
III–IV	12	6	6	
MCAP (mm^2^)	251.7 ± 61.9	242.7 ± 45.0	258.2 ± 72.0	0.467
Anesthesia duration (h)				0.418
≤2.5	22	8	14	
>2.5	14	7	7	
Recovery time (h)				0.607
≤8	15	7	8	
>8	21	8	13	
LSAT (%)	85.4 ± 7.0	88.5 ± 2.9	83.1 ± 8.3	0.010*
ASAT (%)	94.5 ± 1.8	94.5 ± 1.6	94.5 ± 2.0	0.988
ODI4 (events/h)	10.9 ± 10.8	8.1 ± 7.4	12.9 ± 12.5	0.188
CT90 (%)	5.6 ± 14.0	2.4 ± 4.3	7.8 ± 17.8	0.197

LSAT, lowest oxygen saturation; BMI, body mass index; MCAP, minimal cross-sectional area of the pharynx; ASAT, average oxygen saturation; ODI4, oxygen desaturation index of ≥4%; CT90, and percentage of time with oxygen saturation below 90%.

* Statistically significant.

A logistic regression analysis that studied all the variables in Table 2 to predict ΔLSAT was performed. As shown in Table 3, BMI, LSAT, and modified Mallampati grade were found to be independently predictive for ΔLSAT (all *P*’s < 0.05).

**Table 3:** Independent prediction variables for ΔLSAT.

	OR value	95% CI	*P* value
BMI (kg/m^2^)	1.492	1.034–2.151	0.032*
LSAT (%)	1.638	1.125–2.386	0.010*
Modified Mallampati grade	3.141	1.054–9.364	0.040*

LSAT, lowest oxygen saturation; BMI, body mass index.

* Statistically significant.

## Discussion

In patients who underwent bilateral nasal packing after septoplasty and/or functional endoscopic sinus surgery, we found that the overall sleep hypoxemia severity deteriorated during the first postoperative night, compared with preoperative conditions. The numbers of patients with sleep hypoxemia and moderate-to-severe sleep hypoxemia increased during this night, which were similar to the results of some previous studies (7, 8). Postoperative hypoxemia after general anesthesia is a predictor of increased risks of cardiopulmonary complications (9–11). Therefore, in patients undergoing bilateral nasal packing, alternative methods, such as trans-septal suture, that had less impact on sleep breathing should be recommended to reduce the perioperative risks associated with hypoxemia, especially in those with high risks of cardiopulmonary complications (4).

The mechanisms for the above results are considered as follows: the first is the accumulation effect of anesthetics, as it could reduce upper airway muscle tension and increase arousal threshold a few days after the operation, especially the first postoperative night, and could also interfere the stability of respiratory regulation during this period (12–14); the second is the disappearance of nasal ventilatory reflex, as respiratory airflow could not activate nasal mucosal receptors to stimulate respiratory reflex, which could interfere with the spontaneous ventilatory response and further reduce upper airway muscle tension (15); and the third is the unstable transoral breathing, as compared with transnasal breathing, oral breathing could cause some anatomical changes, including backward movements of the tongue, increased pharyngeal compliance, and soft palate oscillations, which can promote the occurrence or aggravation of sleep-disordered breathing and associated hypoxemia (16, 17).

LSAT is the most common index to determine the severity of sleep hypoxemia. We further found that BMI, LSAT, and modified Mallampati grade are independent predictors of ΔLSAT, suggesting that patients with obesity, relatively normal sleep oxygen saturation, and high modified Mallampati grades are more likely to present a larger drop in sleep oxygen saturation on the night after general anesthesia. Therefore, more attention should be paid. The following reasons are considered. Regarding BMI, obese patients are speculated to have a great accumulation of anesthetic drugs. Patients with lower preoperative LSAT are more likely to have oral breathing habits because of higher possibilities of sleep-disordered breathing. Therefore, the decrease in LSAT related to the change in respiratory habits then may be smaller. Friedman et al. found that compared with patients with moderate-to-severe obstructive sleep apnea syndrome (OSAS), those with mild disease were more likely to have increased OSAS severity after nasal packing (18). Regli et al. found that non-OSAS patients were more likely to have increased sleep hypoxemia with nasal packing after general anesthesia (19). The clinical implication is that although OSAS is an independent predictor of increased risks of complications after general anesthesia (11, 20–22), patients with non-OSAS should also be paid attention after bilateral nasal packing because of the high possibility of great deterioration of sleep hypoxemia after general anesthesia. Patients with high modified Mallampati grades are more likely to have glossopharyngeal collapse during sleep (6). In the presence of reduced upper airway tone due to anesthetic accumulation, glossopharyngeal obstruction is more likely to occur in patients with high modified Mallampati grades during the first postoperative night, which could cause a great decrease in sleep oxygen saturation.

In this study, no significant associations were found between anesthesia duration and recovery time with ΔLSAT. Except for a relatively small sample size, the short duration of this kind of surgery may also be one of the reasons. Nevertheless, we still recommend that patients who undergo bilateral nasal packing after general anesthesia should minimize anesthesia time and prolong recovery time by scheduling the surgery early in the day.

The limitations of this study were as follows: first, the sample size is relatively small; however, we still got the expected results. Further research that could include a greater sample size may help verify current conclusions. Second, we did not use a more accurate test, such as polysomnography, to evaluate the severity of sleep hypoxemia and sleep-disordered breathing. This may lead to a certain bias in the evaluation. However, the pulse oximetry test is simple and causes little interference with normal sleep, which could minimize the possibility of patient withdrawal. At last, no control group was included, such as patients who received trans-septal suture management. It may be helpful to further investigate the effect of bilateral nasal packing on sleep oxygen saturation by a stricter study design in the future.

## Conclusion

In summary, we found that bilateral nasal packing after general anesthesia could induce or aggravate sleep hypoxemia, especially in patients with obesity, relatively normal sleep oxygen saturation, and high modified Mallampati grades. Therefore, more attention should be paid to such patients, especially to those with high risks of cardiopulmonary complications.

## Data Availability

The original contributions presented in the study are included in the article/Supplementary Material; further inquiries can be directed to the corresponding authors.
